# Efficacy of polyethylene glycol in the treatment of spinal cord injury and its effect on inflammatory response and oxidative stress factors

**DOI:** 10.3389/fneur.2025.1715518

**Published:** 2025-12-17

**Authors:** Jia-Yang Chen, Jia-Xing Wang, Wei-Hua Zhang, Xiao-Ping Ren

**Affiliations:** 1 School of Graduate, Guangxi University of Chinese Medicine, Nanning, China; 2 Department of Orthopedics, Ruikang Hospital Affiliated to Guangxi University of Chinese Medicine, Nanning, China; 3 School of Medicine, Guangxi University, Nanning, China

**Keywords:** inflammatory response, mouse, oxidative stress, polyethylene glycol, spinal cord injury

## Abstract

**Introduction:**

Spinal cord injury (SCI) is a severe condition characterized by neuroinflammation and oxidative stress, which hinder neurological recovery. Polyethylene glycol (PEG) has shown multiple therapeutic benefits in SCI, such as repairing axonal membranes, improving the post-injury microenvironment, preventing nerve fiber degeneration, and inhibiting spinal cord vacuolation and scar formation. Based on these properties, PEG is regarded as a potential fusogen capable of promoting functional recovery after SCI. This study aimed to further investigate the effects of PEG on SCI and elucidate its underlying molecular mechanisms using a mouse spinal cord total transection model.

**Methods:**

A mouse model of complete spinal cord transection was established to evaluate the therapeutic potential of PEG. Motor function recovery was assessed using the Basso Mouse Scale (BMS) and footprint analysis. Oxidative stress levels were measured via superoxide dismutase (SOD) and malondialdehyde (MDA) assay kits, while inflammatory cytokine expression was analyzed using enzyme-linked immunosorbent assay (ELISA). Histopathological examination was performed to evaluate axonal and myelin preservation and cystic vacuole formation. Immunofluorescence staining was used to observe axonal regeneration and neuroprotection. Additionally, electrophysiological tests were conducted to assess the recovery of nerve conduction.

**Results:**

Mice treated with PEG showed significantly improved BMS scores at 7, 14, and 28 days post-injury compared to the untreated SCI group, indicating enhanced motor function recovery. Biochemical assays revealed that PEG markedly reduced oxidative stress and suppressed the expression of pro-inflammatory cytokines during the early phase of SCI. Histopathological analysis demonstrated that PEG treatment protected spinal cord axons and myelin tissue and significantly reduced the formation of cystic vacuoles. Immunofluorescence results indicated that PEG exerted neuroprotective effects and promoted axonal regeneration after SCI. Electrophysiological assessments further confirmed improved recovery of nerve conduction in PEG-treated mice.

**Discussion:**

The findings of this study demonstrate that PEG, as a fusogen, exhibits significant neuroprotective and regenerative effects when applied immediately after SCI. PEG not only attenuated oxidative stress and neuroinflammation but also preserved axonal integrity, promoted myelin protection, and enhanced functional recovery. These results suggest that early application of PEG represents an innovative and promising therapeutic strategy for SCI, warranting further investigation into its long-term efficacy and potential clinical translation.

## Introduction

SCI is a serious injury to the central nervous system that can lead to motor, sensory, and autonomic dysfunction, which severely reduces the quality of life of patients (1). The annual incidence of SCI is reported to be 10.4 − 83/100,000 worldwide, which has so far resulted in lifelong disability for about 27 million people worldwide, imposing a heavy economic burden on both families and society (2, 3). The pathogenesis of SCI includes primary injury and secondary injury. Primary injuries are mainly caused by spinal cord trauma or violence and are related to the destruction of axons and neurons. Secondary injury includes a number of complex phenomena such as increased cell permeability, lipid peroxidation, neuroinflammation, oxidative stress, demyelination, Wallerian degeneration, fibroglial scarring, and cyst formation, which further induce severe tissue damage and inhibit axonal regeneration in the diseased region (4, 5). Unfortunately, the spinal cord is particularly vulnerable and sensitive to these deleterious factors, resulting in tissue necrosis and functional deficits. Due to the complexity of the central nervous system, the underlying mechanisms of SCI remain unclear, and to date, no effective therapeutic agents or other interventions have been identified to effectively inhibit the development of secondary injuries after SCI and to effectively promote the recovery of motor function.

PEG a synthetic biocompatible polymer with the ability to “fuse” transected neuronal cell bodies and nerve fiber membranes, has made some progress in the treatment of SCI (6). The direct application of polyethylene glycol as a mitogen to the site of injury can repair cell membranes, reduce oxidative stress, promote axonal regeneration, and restore motor function (7, 8). Polyethylene glycol can also covalently or non-covalently bind to proteins, peptides, and nanoparticles to limit their clearance by the reticuloendothelial system, reduce their immunogenicity, and facilitate the crossing of the blood–brain barrier, which is widely used as a drug carrier (9, 10). Crosslinked polyethylene glycol produces hydrogels that can be used as delivery vehicles for bioactive molecules, including growth factors and cells such as bone marrow stromal cells, which can modulate inflammatory responses and support the regeneration of neural tissues. At the same time, polyethylene glycol hydrogels separate or reduce localized glial scarring invasion, promote and guide axonal regeneration across the grafted area, and re-establish synaptic connections with target tissues, thereby promoting spinal cord repair (11, 12). Based on these studies, we believe that polyethylene glycol is a promising synthetic material for SCI repair. In 2013, our research team, in collaboration with international experts, introduced the PEG-mediated Spinal Cord Fusion (SCF) technique. This technique utilizes a sharp blade to “cleanly cut” the spinal cord to form two tightly aligned segments that are immediately fused locally with PEG (13). It has been applied to a variety of animal models, including mice, rats, beagles, and monkeys (14–18), and studies have shown that SCF exhibits varying degrees of functional recovery of the hindlimb at the T10 level after acute SCI.

However, while the potential benefits of PEG in improving behavioral recovery in animal models of SCI have been suggested in previous studies, in-depth studies on its specific mechanisms of action remain insufficient. Particularly in the fields of histopathology and immunology, previous studies have failed to provide sufficient direct evidence to support these findings. The present study builds on previous work with an extended analysis of the behavioral, histological, and immunological aspects of PEG in a mouse model of a fused transected spinal cord. We found also that PEG was able to attenuate oxidative stress and inflammatory responses after SCI and restoration of spinal cord electrical signaling. These findings provide deeper evidence for the use of PEG in promoting neuroregeneration and repair after SCI, thus enhancing the SCF technology as a practical therapeutic option and providing additional theoretical support for the clinical translation of SCF.

## Methods and methods

### Animal

The experimental animals were 60 healthy female C57BL/6J mice, weighing (20 ± 2 g), aged 6–8 weeks, purchased from Changsha Tianqin Biotechnology Co., LTD. [SCKX (Xiang) 2022-0011], and fed in the animal room of the Clinical Medical College of Guangxi University of Traditional Chinese Medicine [SYXK(GUI)2019-0001]. Female mice are used to be the experiment model because of a higher incidence of urinary retention in male rats after SCl. Temperatures ranged from 22 °C to 25 °C, and all mice were given 12-h light/dark cycle exposure with water and feed AD libitum.

### Ethics statement

The protocol was rigorously reviewed and approved by the Institutional Animal Care and Use Committee of the Guangxi University of Chinese Medicine (DW20230525-089).

### Spinal cord injury model and drug treatment

In this study, adaptive feeding mice for 1 week were randomly divided into the Sham group, SCI group, and SCI + PEG group (*n* = 20 each). All animals were anesthetized by inhalation of 3% isoflurane, immobilized in the supine position on a thermostatic heating plate to maintain body temperature, and then kept anesthetized by continuous inhalation of 1.5% isoflurane. This was followed by back shaving, disinfection with iodine and alcohol, an incision made at T10, and laminectomy. In the sham-operated group, only the spinal cord was exposed without transection. In the SCI group and SCI + PEG group, the T10 laminectomy was removed and the spinal cord was transected with an ultra-sharp microsurgical blade. Peg-600 (100%, Sigma-Aldrich/Merck) (0.2 mL) was immediately applied to the PEG group and administered via a syringe. Stump-end fusion was performed at the transection site followed by layer-by-layer suturing. After the operation, the mice were divided into groups, and artificial urination was performed twice a day (morning and evening), and penicillin (400,000 units/kg) was injected subcutaneously once a day for 7 consecutive days.

### Tissue preparation

Ten mice from each group were sampled on postoperative day 3 for SOD activity and MDA content measurement and Elisa assay. The mice were anaesthetized with 10% isoflurane to induce deep anaesthesia to avoid pain, and the limbs were fixed with needles and placed in supine position on the operating table for cardiac perfusion with saline. Immediately after perfusion, 0.5 cm of tissue was removed from the top and bottom of the severed end of the spinal cord of the mice and stored at −80 °C for subsequent experiments.

Spinal cord samples from the remaining 10 mice in each group were obtained in the same way on day 28 postoperatively for pathology analysis. But, after the completion of saline perfusion, 4% paraformaldehyde was continued until the perfusion was stopped after muscle contraction and upward arching of the thorax were observed in the mice. Similarly, 0.5 cm above and below the severed end of the spinal cord was also taken, fixed with 4% paraformaldehyde for 24 h, then embedded in paraffin wax and sectioned at 4 μm for subsequent histopathological evaluation and immunohistochemical staining.

### Locomotion recovery assessment

Mice’s hindlimb motor function was evaluated using the BMS scores, and footprint analysis. Test time points were established at days 0, 1, 3, 7, 14, 21, and 28, with footprint analysis conducted on day 28. BMS scoring system ranges from 0 to 9, where 0 indicates complete paralysis and 9 signifies normal function. This assessment was based on the frequency of observations, range of motion, coordination, dorsiflexion of the ankle joint, tactile sensation on the plantar and dorsal surfaces of the foot, foot positioning, trunk stability, and tail position. For the footprint analysis, the forepaws and hindpaws of each animal were coated with blue and red dye, respectively. Two evaluators, who were unaware of the experimental conditions and had received specialized training, conducted the assessments in triplicate and recorded the results immediately.

### Electrophysiological examination

Four weeks post-surgery, electrophysiological assessment was conducted using the NIM-ECLIPSE® system (Medtronic, USA), with a particular focus on motor-evoked potentials (MEPs). Transcranial electrical pulses with a pulse width of 75 μs and a voltage of 10 V were employed to elicit MEPs during anodal stimulation training. The stimulation electrodes were precisely positioned over the C3 and C4 motor cortical areas, following the international 10–20 EEG system. Meanwhile, recording electrodes were strategically placed over the tibialis anterior muscle of the lower limbs.

### HE and LFB staining

Sections were cut (4 μm thick) from 4% paraformaldehyde-fixed and paraffin-embedded spinal cord segments and were prepared for histological staining at 28 days after SCI. For HE staining, sections were stained with HE reagent (Servicebio, China, G1076) according to the manufacturer’s instructions and viewed with a microscope (Nikon Eclipse E100, Japan). For LFB staining, the sections were treated with myelin staining solution (Servicebio, China, G1030), washed with water to terminate differentiation, microscopic examination. This process was repeated until the myelin sheath was blue, and the background was almost colorless. Subsequently, the sections were counterstained with eosin (Servicebio, China, G1002), dehydrated, and mounted. The prepared slides were observed under a microscope (Nikon Eclipse E100, Japan), and images were captured for further analysis.

### SOD activity assay and MDA assay

The activity of SOD and MDA levels in the spinal cord were measured using the Xanthine Oxidase Kit (Jiancheng, China, A001-1) and the Lipid Peroxidation MDA Kit (Servicebio, China, G4300-48T), respectively. On day 3 after SCI, spinal cord tissue was harvested following the manufacturer’s instructions. The SOD activity in the samples was determined by spectrophotometry at 550 nm, which measures the absorbance of the color produced by the reaction of the xanthine oxidase system and SOD. The MDA levels were assessed by measuring the absorbance at 532 nm, which is the color produced by the reaction between thiobarbituric acid and malondialdehyde.

### Measurement of inflammatory factor levels by ELISA

At 3 days after surgery, spinal cord tissues from each group were harvested. After grinding and centrifugation to obtain the supernatant, the samples were diluted with standard diluent as per the instructions of the ELISA kits for TNF-α (Rui Xin, China, RX202412M), IL-1β (Rui Xin, China, RX203063M), and IL-6 (Rui Xin, China, RX203049M). Following the addition of the stopping solution and mixing, the samples were analyzed using a microplate reader to measure the levels of inflammatory cytokines TNF-α, IL-1β, and IL-6 in the spinal cord tissues of each group.

### Immunofluorescence staining

The corresponding spinal cord tissues were also obtained on day 28 after SCI. Then, the paraffin sections of the tissue, which had been dehydrated in an absolute ethanol gradient, were fully blocked with 3% bovine serum albumin (BSA) for 30 min at room temperature. After adequate blocking, slides were washed correctly three times with phosphate buffered saline (PBS) solution and incubated with appropriate primary antibodies overnight at 4 °C in the same buffer. The following primary antibodies were used: NF-200 (1:500), MBP (1:200), NeuN (1:500), and 5-HT1A (1:500), col-1 (1:300), CNPase (1:300), Synaptic (1:200), all from Servicebio. These markers are integral for assessing neural integrity and function post-SCI. Overnight sections were incubated with Alexa Fluor 488-labeled Goat Anti-Mouse IgG (H + L) conjugated secondary antibody for 50 min at room temperature, nuclei were counterstained with DAPI, and anti-fluorescence quenched sealing tablets were sealed. Spinal cord sections were imaged by fluorescence microscopy (Nikon, Japan).

### Statistical analysis

The statistical analysis was conducted using SPSS 20.0 (IBM Inc., SPSS Statistics, Armonk, NY, USA) and GraphPad Prism 8 (GraphPad Software, La Jolla, CA, USA) for both statistical analysis and graphical representation purposes. The results are presented as the means ± standard deviation (SD). Intergroup comparisons were performed using one-way analysis of variance (ANOVA), repeated-measures ANOVA, and Tukey’s *post hoc* test. Each experiment was performed three times independently to ensure the reliability of the experimental data. The *p* values <0.05 indicated statistical significance.

## Results

### PEG improves functional recovery after SCI

**Figure 1:**
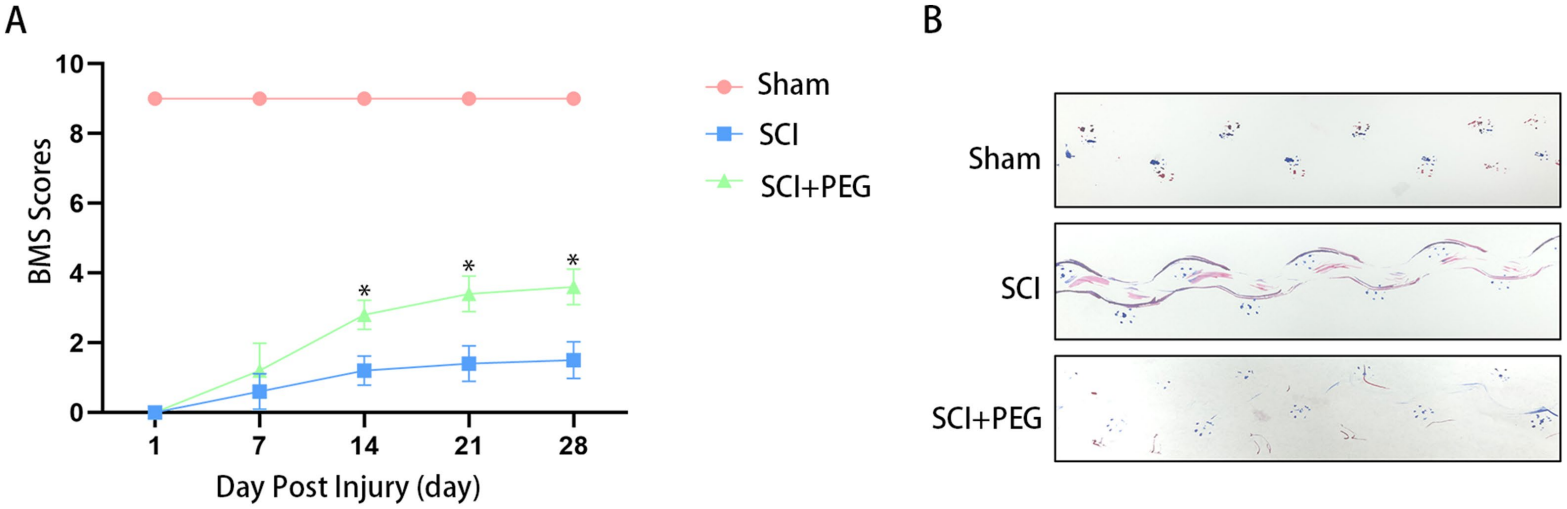
PEG improves functional recovery after SCI. **(A)** BMS scores at different time points after SCI in each group: compared with the SCI group, the behavioral scores in the PEG group were significantly higher than those in the SCI group (*p <0.05). *n* = 10/group. All data are presented as mean ± SD. Statistical analysis was performed with repeated-measures ANOVA. **(B)** The results of footprint analysis in each group 28 days after SCI (the front and back paws of mice were stained with blue and red dyes, respectively).

Behavioral experiments, including BMS score and footprint analysis, were performed to evaluate functional recovery after SCI. The results showed that compared with the Sham group, the BMS scores of both groups were 0 at the early stage after SCI (1, 3, and 7 days). However, compared with the SCI group, the PEG treatment group obtained better BMS scores at 14, 21, and 28 days(average BMS subscores of 3.4 ± 0.52 and 3.6 ± 0.52 for 21 and 28 days animals respectively) (Figure 1A). Similarly, the therapeutic effect was validated by footprint analysis at 28 days. The PEG-treated group showed coordinated crawling of the hind limbs (red dye) and a slight waddy gait, whereas the SCI group showed inconsistent behavior, dragging the hind limbs and inking on both fore and hind limbs (marked with blue and red dye, respectively) (Figure 1B). The above results suggest that PEG contributes to functional recovery after SCI.

### PEG improves the pathological injury of SCI mice’s spinal cord

**Figure 2:**
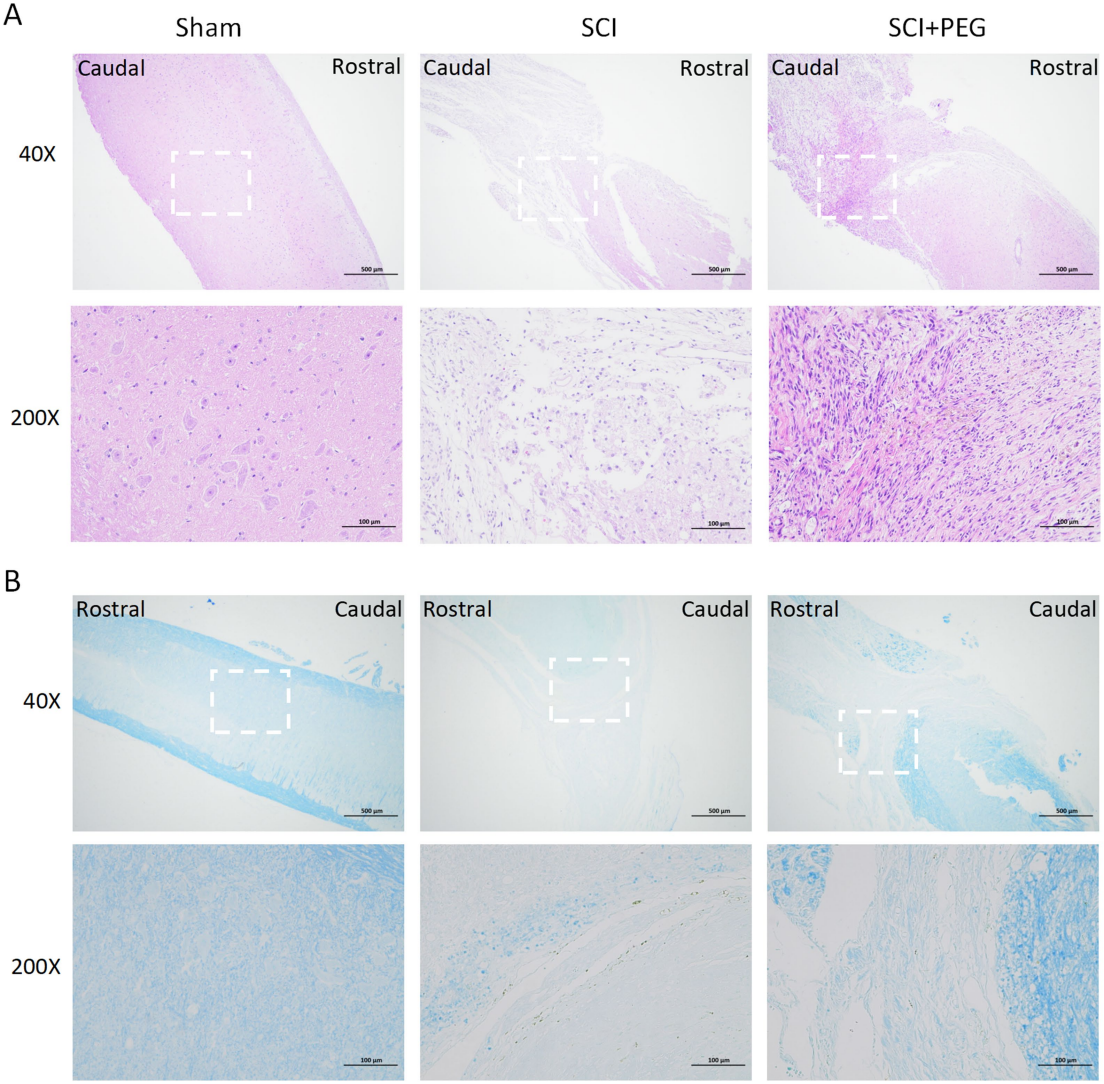
PEG improves the pathological injury of SCI mice spinal cord. **(A)** Results of HE staining of a longitudinal section of the spinal cord in each group 28 days after SCI. *n* = 5/group. **(B)** Results of LFB staining of a longitudinal section of the spinal cord in each group 28 days after SCI. The deeper the staining result, the more complete the myelin structure. *n* = 5/group. Scale bar: 500 μm (inset: higher magnification; scale bar: 100 μm).

HE staining and LFB staining were used to evaluate the effect of PEG on tissue structure and morphology and myelin regeneration local feedback staining. HE staining showed (Figure 2A) that the spinal cord tissues in the Sham group were structurally intact and neatly arranged, and the morphology of nerve cells was normal. A larger number of nerve fibers were seen in the nerve tissues of the SCI group, which were swollen, vacuolated, and irregularly arranged, and necrotic dissolution of the nerve fibers was rare, with hemorrhage seen locally; no obvious inflammatory cell infiltration was seen. In the SCI + PEG group, a decrease in the number of nerve fibers was seen in the nerve tissue, a larger number of macrophage infiltration was seen, and connective tissue hyperplasia was seen locally. Tissue damage and cavity formation were significantly reduced in the treated group compared to the SCI group.

Consistent with this view, LFB staining also showed (Figure 2B) that the SCI group exhibited severe myelin destruction and myelin loss, in contrast to the PEG-treated group, which significantly ameliorated these deficits. Under LFB staining, the nerve fiber myelin sheaths of the Sham group were stained blue, and the nerve myelin sheaths were normal; a greater number of demyelinated nerve fibers were seen locally in the neural tissues of the SCI group; a greater number of demyelinated nerve fibers, but with a greater amount of connective tissue infiltration, were seen locally in the neural tissues of the SCI + PEG group. More nerve fibers were demyelinated in the SCI + PEG group but less than in the SCI group. Taken together, these findings suggest that PEG helps to restore tissue structure and morphology and regenerate myelin sheath after injury, thus improving functional recovery after SCI.

### PEG alleviates oxidative stress and inflammation after SCI

**Figure 3:**
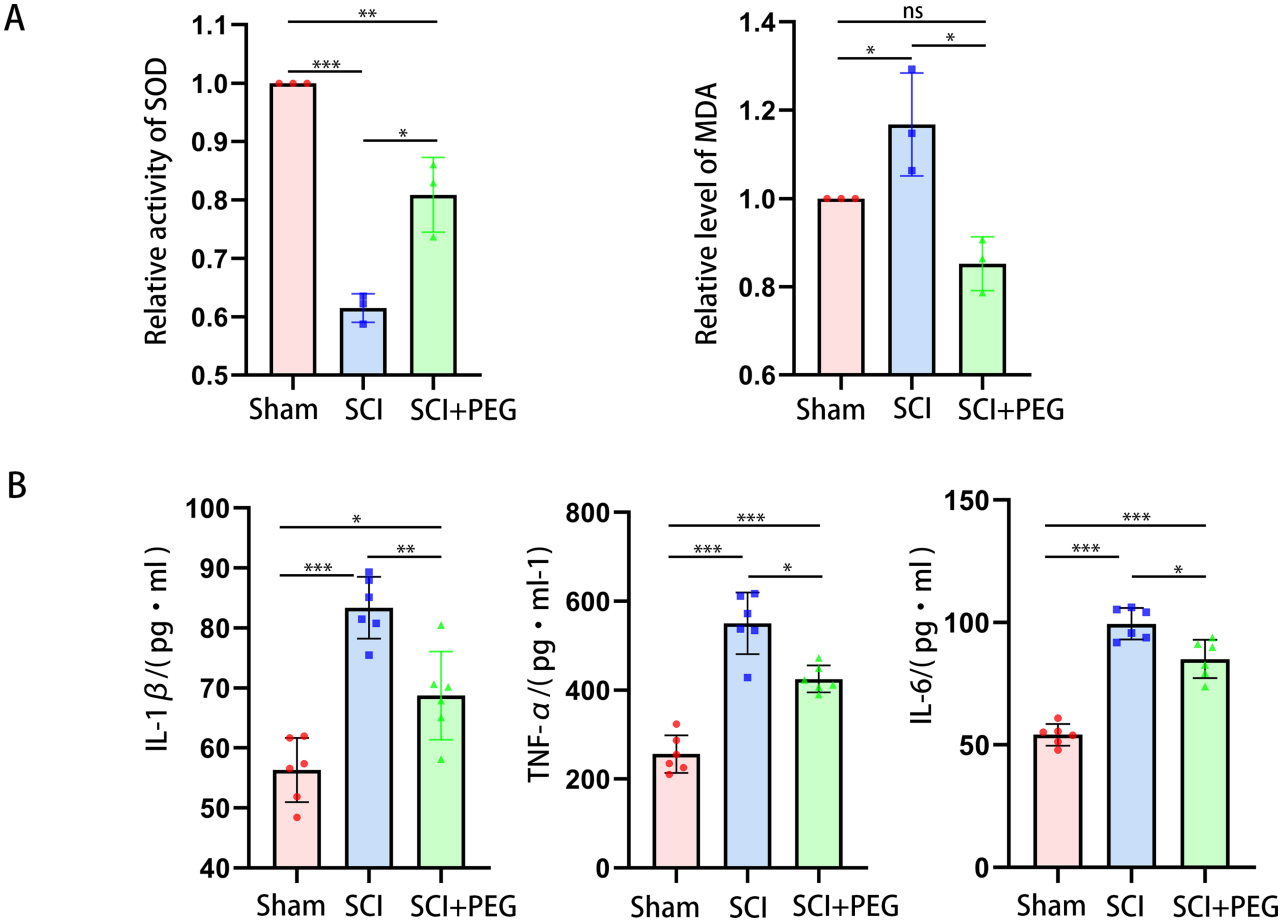
PEG alleviates oxidative stress and inflammation after SCI. **(A)** The activity of SOD and MDA levels in spinal cord tissues in the different groups. *n* = 3/group. (*p < 0.05; **p < 0.01; ***p < 0.001). **(B)** Representative images of B displayed the level of IL-1β, IL-6, and TNF-α in the spinal cord. *n* = 6/group. (*p < 0.05; **p < 0.01; ***p < 0.001). All data are presented as mean ± SD. Statistical analysis was performed with one-way ANOVA and Kruskal–Wallis H test.

The results are shown in Figure 3A. Compared with the Sham group, the content of MDA in the spinal cord tissue of the SCI group was significantly increased, while the activity of SOD was significantly decreased. Compared with the model group, the content of MDA in the spinal cord tissue of the SCI + PEG group was significantly decreased, while the activity of SOD was significantly increased. It is suggested that PEG can reverse the imbalance of MDA and SOD and reduce oxidative damage after spinal SCI. In addition, we found that PEG could inhibit the levels of IL-1β, IL-6, and TNF-α in the spinal cord of the injured mice (Figure 3B), indicating an anti-inflammatory effect.

### PEG promotes axonal regeneration after SCI

**Figure 4:**
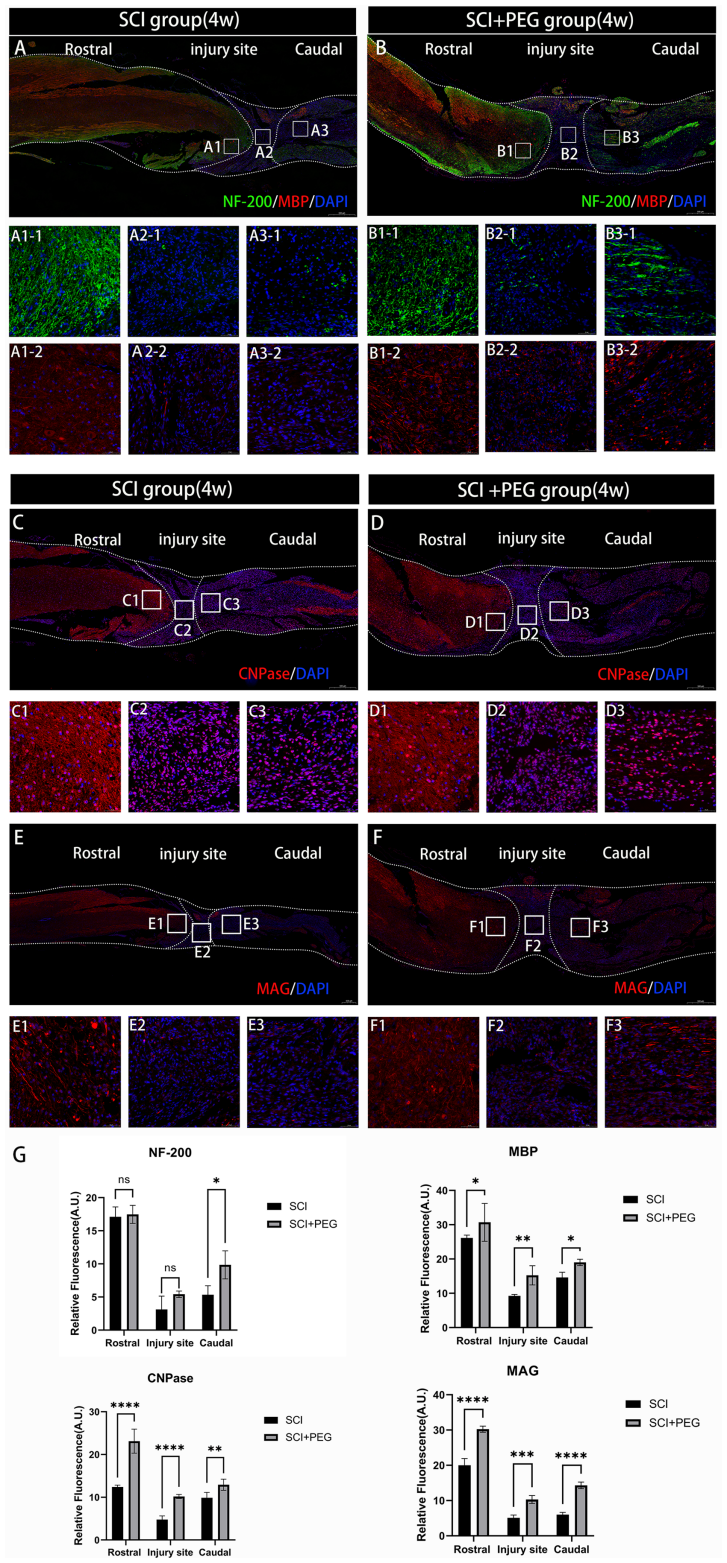
PEG promotes axonal regeneration after SCI. **(A,B)** Double-fluorescence staining analysis for NF-200 (green)/MBP (red) in the injured spinal cord. **(C–F)** Immunofluorescence method to observe CNPase and MAG positive expression in the spinal cord of mice in SCI group and SCI + PEG group. The stronger the fluorescence response, the higher the protein expression. *n* = 5/group. Scale bar: 500 μm (inset: higher magnification; scale bar: 50 μm). **(G)** Expression trends of NF-200, MBP, CNPase and MAG in spinal cord tissues stained by immunofluorescence. *n* = 3. (*p < 0.05; **p < 0.01; ***p < 0.001; ****p < 0.0001).

Immunofluorescent staining of spinal cord tissue sections with anti-NF-200 antibody-labeled neurofilament (green) and anti-MBP antibody-labeled myelin basic protein (red). Our data showed that very few neurofilaments survived in the injured area in the SCI group at 28 d after SCI (Figure 4A), and the application of PEG treatment significantly enhanced the expression of NF-200 and prevented severe loss of neurofilaments after SCI. In addition, the expression of MBP was also significantly enhanced after treatment with PEG, exerting a neuroprotective effect (Figure 4B). In addition, the expression of oligodendrocyte markers CNPase, myelin-associated glycoprotein MAG, which is also important for axonal regeneration. As shown in the graphs (Figures 4C–G), the fluorescence intensity of CNPase and MAG was significantly increased after applying PEG treatment to SCI compared with the SCI group. Taken together, these results suggest that PEG has a neuroprotective effect on axonal regeneration after SCI.

### PEG promotes nerve conduction recovery after SCI

**Figure 5:**
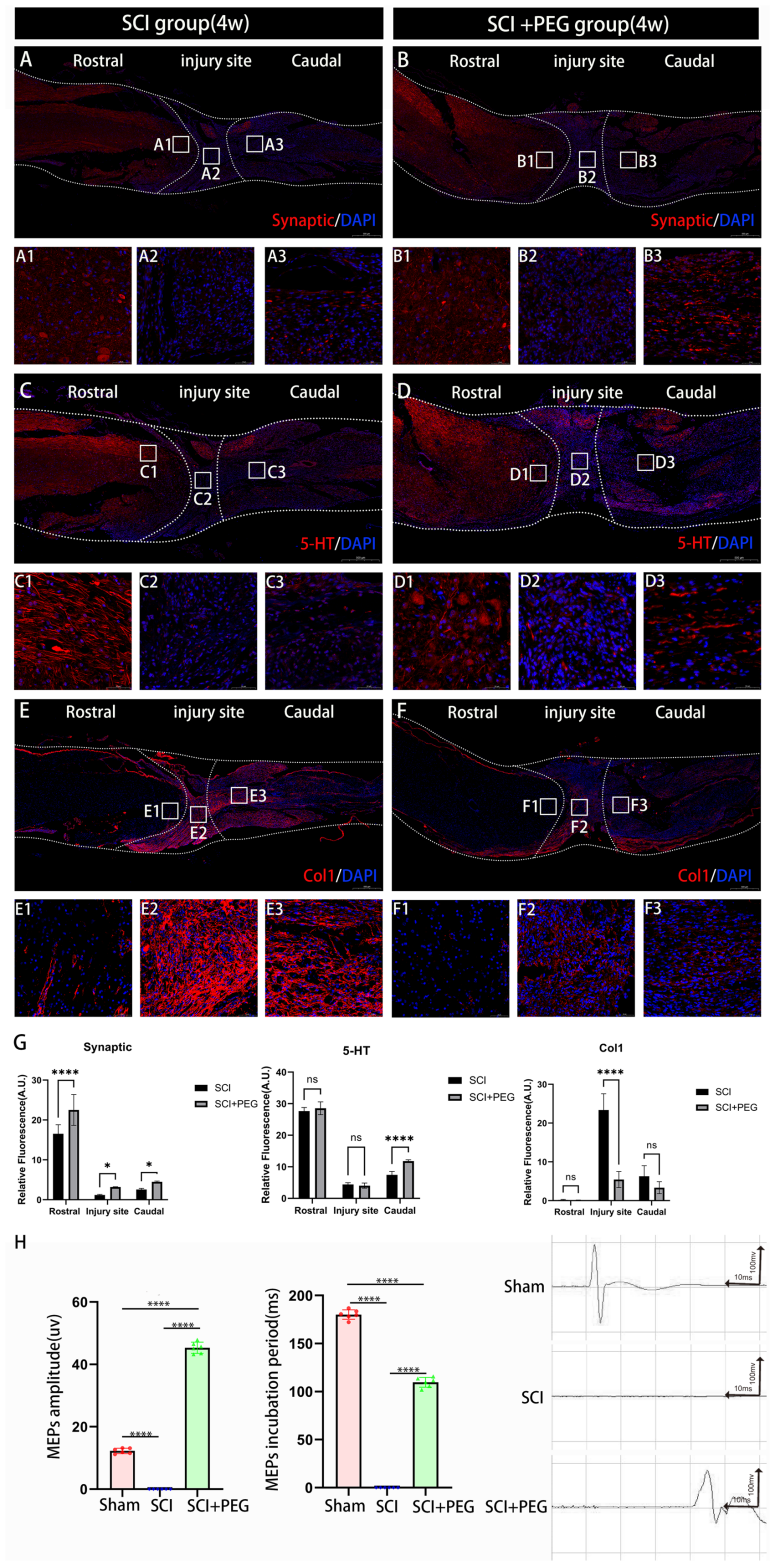
PEG promotes nerve conduction recovery after SCI. **(A–F)** Immunofluorescence method to observe Synaptic, 5-HT and Col-1 positive expression in the spinal cord of mice in SCI group and SCI+PEG group. The stronger the fluorescence response, the higher the protein expression. **(G)** Expression trends of Synaptic, 5-HT and Col1 in spinal cord tissues stained by immunofluorescence. *n* = 3. (*p < 0.05; **p < 0.01; ***p < 0.001). **(H)** Results of electrophysiological assessment in each group at 28 days after SCI. (****p < 0.0001). *n* = 6/group. All data are presented as mean ± SD. Statistical analysis was performed with one-way ANOVA and Kruskal–Wallis H test. Scale bar: 500 μm (inset: higher magnification; scale bar: 50 μm).

To further determine the effect of PEG on signaling *in vivo*, we used immunofluorescence staining to detect the expression of Synaptic, 5-HT and Col-1 in the SCI region to assess the effect of PEG on signaling after SCI. As shown in Figures 5A–D, the fluorescence intensity of Synaptic and 5-HT was significantly increased in the PEG-treated group compared with that in the SCI group, and both of them were related to the transmission and regulation of nerve signals. Another protein Col-1, although not a direct neurotransmitter, its expression reflected the scar formation after SCI (19), and we found that the fluorescence intensity of Col-1 decreased significantly after the application of PEG (Figures 5E–G). These activated astrocytes form a glial scar in the injured area, which may hinder neuronal regeneration and axonal regeneration. Electrophysiological results showed (Figure 5H) that the waveforms collected in the SCI + PEG group were similar to those in the Sham group, with a longer latency, whereas the waveforms could not be detected in the SCI group. Taken together, our results suggest that PEG promotes the recovery of MEP signaling in the lower limbs.

## Discussion

SCI disrupts motor, sensory, and autonomic functions, which severely reduces patients’ quality of life and imposes a heavy economic burden on families and society. A total of 759,302 cases of traumatic SCI have been reported in China, with 66,374 new cases occurring each year (20, 21). In addition, data from the United States show that the incidence of SCI is about 17,000 per year, and the first-year cost of a patient with severe quadriplegia is more than 1 million USD (22). Most of the spinal cord tissue injuries occurring after SCI are exacerbated by secondary injuries. Therefore, prevention of secondary injuries in the early stages of SCI is imperative.

In this study, we investigated the effects of PEG on oxidative stress and neuroinflammation after SCI. First, after mice underwent a complete spinal cord transection model and were immediately given PEG treatment, we examined SOD activity and MDA levels at the SCI site and found that the SOD and MDA contents in the SCI group were significantly higher than those in the SCI + PEG group. In the normal physiological state, these substances maintain a dynamic balance due to the removal of necrotic cells and metabolic wastes. However, in the injured state, this balance is disrupted and intracellular hypercalcemic accumulation leads to neuronal excitotoxicity, increased reactive oxygen species concentrations and glutamate levels and oxidative stress (23, 24). These occurrences impair underlying nucleic acids, proteins, and phospholipids and lead to neurological dysfunction. These findings suggest that immediate activation of cellular anti-oxidative stress mechanisms should provide protection against irreversible tissue damage and its far-reaching detrimental effects on SCI-associated motor function. PEG helps to achieve a state of homeostasis in the local microenvironment and accelerates the clearance of ROS by promoting SOD activity and decreasing MDA levels, thus preventing further cellular damage.

Furthermore, in traumatic injuries to the central nervous system, the early inflammatory response has a significant impact on injury prognosis (25). A complex series of pathological events occur successively during the secondary injury phase after SCI, where the injury leads to rupture of blood vessels, causing hemorrhage in the spinal cord tissue, followed by invasion of monocytes, neutrophils, T and B lymphocytes, and macrophages into the spinal cord tissue (5, 26, 27). This process is also associated with the release of inflammatory cytokines, such as interleukins IL-1a, IL-1b, IL-6, and tumor necrosis factor TNF-α, 6 ~ 12 h after injury (28, 29). Infiltration of immune cells and inflammatory cytokines promotes neuronal inflammation, induces neuronal apoptosis, spreads inflammation to neighboring tissues, causes ROS release and cell death, and limits tissue regeneration and functional recovery (30). After PEG treatment, we examined the expression of the early inflammatory factors IL-1β, IL-6, and TNF-α, and we were surprised to find that the levels of these inflammatory factors appeared to be reduced to some extent in SCI mice immediately after PEG treatment was given. Therefore, we hypothesized that PEG may protect peripherally damaged neurons by suppressing the inflammatory response after SCI.

Early apoptosis and autophagy after SCI have a protective effect on the organism, prolonged inflammatory response leads to accumulation of proinflammatory factors and excessive apoptosis in peripheral normal neuronal cells, as well as excessive autophagy, which exacerbates TSCI during the subacute phase of the secondary injury. Initial glial gelatinous scarring during the subacute phase is a protective mechanism against TSCI, which restricts the spread of injury and inflammation. However, mature scar tissue hinders axonal regeneration in the chronic phase and prevents recovery of nerve function. Failure of axonal regeneration is generally recognized as a major obstacle to recovery from SCI, the main cause of nerve injury. To further reinforce the idea that PEG can promote axonal myelin regeneration after SCI, we also examined the expression of the oligodendrocyte-specific markers CNPase and the transmembrane glycoprotein MAG, both of which showed an increase. Elevated levels of CNPase provide favorable conditions for the proliferation and differentiation of oligodendrocytes, which are essential for repairing damaged spinal cord tissue (31). MAG is expressed in the axonal membrane of oligodendrocytes between the axon and the inner myelin sheath, which functions to maintain myelinated axons in the adult nervous system; therefore, a decrease in MAG in the SCI model may indicate damage and detachment of myelinated axons (32). In addition, recovery of hindlimb motor function in SCI is an important indicator of axonal regeneration, therefore, we used a series of behavioral tests to assess this. At the 4-week evaluation, the PEG-treated mice showed significantly improved recovery of motor function, achieving a BMS score of (3.6 ± 0.52), compared to (1.5 ± 0.53) in the SCI group, a statistically significant difference. In addition, mouse footprint analysis further supported this quantitative result.

Synaptic is a key structure for the transmission of signals between neurons. In SCI, synaptic integrity is critical for neural signaling. Injury may lead to disruption of synaptic structures, which may affect signaling (33). It has been shown that newborn 5-HTergic neurons provide a high concentration of 5-HT microenvironment to the spinal cord segments at the site of injury through their high-intensity and continuously active electrical activity, which can promote the repair of damaged spinal cord neural circuits and the recovery of motor function (34). The decrease in the fluorescence intensity of Col-1 also suggest that the application of PEG can reduce glial scar hyperplasia, promote neuronal regeneration and axon regeneration, and improve spinal cord continuity (35, 36). Electrophysiological data further support this conclusion.

This study also has some limitations. For example, PEG has many biological activities. However, we used only a limited number of markers to study their antioxidant stress and anti-neuroinflammatory effects after SCI, and the sample size was small. Therefore, the other protective effects of PEG on spinal cord injury and its mechanism need to be further studied with a larger sample size. Our future research will focus on elucidating the deeper molecular mechanisms of PEG and exploring its therapeutic potential for other neurological disorders.

## Conclusion

In summary, the results of the present study suggest that oxidative stress and inflammatory processes during SCI can be partially ameliorated by immediate administration of PEG, which significantly corrects the microenvironment of neural tissue repair, reduces the core area of injury, restores signaling in the injured spinal cord, and promotes the recovery of motor function. These findings suggest that the application of PEG may be a promising therapeutic approach for the treatment of SCI.

## Data Availability

The original contributions presented in the study are included in the article/supplementary material, further inquiries can be directed to the corresponding author.
